# Digital peer mentoring in higher education: Results from a qualitative study involving digital part-time nursing students

**DOI:** 10.1016/j.heliyon.2025.e42454

**Published:** 2025-02-07

**Authors:** Hilde Elisabeth Toreid, Berit Harriet Mosseng Sjølie, Sverre Andreassen Bjørbæk, Marcel Köhler

**Affiliations:** Faculty of Nursing and Health Science, Nord University, Norway

**Keywords:** Qualitative study, Nursing students, Online peer mentoring, Digital mentoring

## Abstract

**Background:**

Peer mentoring in nursing education is essential for supporting students’ learning and development processes and reducing university withdrawal rates. Digital mentoring represents a contemporary approach to organising nursing mentoring processes, previously recognised as a practical component in facilitating clinical assessment.

**Aims:**

This study aims to qualitatively examine the impact of digital peer mentoring on nursing students and to review the experiences of their mentors with online mentoring in a part-time nursing program.

**Design:**

A qualitative study employing in-depth, paired interviews and supplementary questionnaires.

**Data sources:**

Data were collected from eight students enrolled in a four-year part-time Bachelor of Nursing program at a university in Norway from 2022 to 2023.

**Review methods:**

Thematic analysis was conducted following the methodology described by Braun and Clarke.

**Results:**

The experiences of students and their mentors were categorised into three themes: ‘Differences in Motivation,’ ‘No Need for Digital Socialising,’ and ‘The Importance of Clarifying Organisational Tasks.’

**Conclusion:**

Digital mentoring proved challenging to sustain for part-time digital nursing students. The study indicates that effective learning through digital mentoring necessitates stringent organisation, such as clear guidelines from the institution or consistent support from educators. Therefore, enhancing digital mentoring among part-time nursing students requires additional facilitation.

## Highlights

• Effective facilitation and support are crucial for adapting students to the online environment in a digital part-time nursing programme.
• Students and mentors preferred an academic focus over online socialising.
• Students and mentors commenced from different starting points, leading to varying motivations and barriers for peer mentoring.
• Mentors felt uncertain and doubted their social skills for professional nursing when students did not respond.
• Students fulfilled their social needs with fellow students and provided support to each other, primarily through social media.

## 1 Background

Peer mentoring is a collaborative educational approach designed to nurture learning processes and expand students' knowledge. Mentoring relationships can foster both personal and professional growth, thereby enhancing overall well-being [1,2]. Higher education institutions implement mentoring programmes to assist students in completing their studies and to prevent or reduce withdrawal rates [3]. Peer mentoring is a relational process in which less experienced students receive support from their more experienced peers [4]. The aims of mentoring can include psychosocial support, academic assistance, or role modelling [5]. Learning processes are initiated within a social institution, where new students are integrated into a community of practice [6]. Peer mentoring can enhance students’ learning outcomes by clarifying the academic curriculum and the social aspects of student relationships ([4]; [2]). Nursing students, in particular, may struggle to adapt to university studies and experience academic stress. Mentoring can significantly alleviate this stress [7]. Furthermore, less experienced students may find peer mentors more approachable and accessible than their faculty teachers [8].

### 1.1 Mentoring in nursing

Mentoring in nursing can be traced back to Nightingale's theories and her practical teaching skills. In recent decades, mentoring in nursing has been utilised to assist students with both academic and clinical assessments [9]. Mentoring in nursing often focuses on four themes: clinical practice, laboratory work, socialisation, and academic support [10]. Although mentors may be assigned in various ways, in nursing, mentors are traditionally assigned to first-year students. Supporting inexperienced first-year students is particularly necessary to help them cope with academic studies and understand their new professional fields [10]; [11]. The mentoring experiences of nursing students have rarely been explored, but they are often integrated with experiences of clinical supervision [12].

### 1.2 Limited online mentoring in nursing programmes

Academic orientation in online learning processes in nursing in Norway is still in its early developmental stages and is growing rapidly due to online opportunities, flexibility, and availability. In an organisational context, online mentoring as a formal programme in nursing is poorly understood, as virtual technology needs to evolve [13]. However, some evidence indicates that peer mentoring is a considerably beneficial experience, although evidence on how peer mentoring is beneficial on measurement scales remains lacking [11]. Peer mentoring can be important for the personal and professional development of both students and mentors [7]. Mentoring programmes are implemented to reduce student withdrawal rates, and universities are encouraged to initiate mentoring programmes. However, guidelines for implementing mentoring programmes in universities remain lacking, and their implementation is therefore adjustable. Only a few universal theoretical frameworks exist for mentoring in education, such as in the field of business [14].

### 1.3 Theoretical framework for online mentoring

Online learning can differ significantly from traditional face-to-face classes due to the pervasive reliance on online technology for distance education [15]. Furthermore, online learning may present certain challenges, such as technical issues or the need for technical support to connect with the programme [16]. The present study involved a programme for part-time digital nursing students at a university in Norway, which was based on an online relational model according to Salmon [17], as the online theoretical framework is new in nursing programmes. Salmon's five-step relational model describes the stepwise evolution of digital learning processes. This model highlights how students need to onboard their study programme, with gaining access and motivation as the first step. The second step involves familiarising students with online technology platforms and tools, and culturally and socially interacting with their learning environment before the third step, which involves exchanging learning information. The fourth step is building knowledge, and the fifth step involves students' responses to their academic understandings and their overall social development. This theoretical perspective outlines the starting point for first-year students in a digital context.

### 1.4 Aim

This study aimed to assess nursing students’ experiences regarding digital peer mentoring.

## 2 Methodology orientation and methods

This study employs a hermeneutic framework to gain a deeper understanding of the experiences of students and mentors in digital mentoring. A blended approach was utilised, involving both qualitative interviews and qualitative document analysis, with thematic analysis employed as a reflective research practice. Braun and Clarke [18] have affirmed thematic analysis as a flexible methodology, rather than a delimited one, providing greater multiplicity for achieving methodological integrity. Triangulating data from interviews with documents offers multiple sources of evidence for credibility by corroborating the findings. Document analysis is used for systematic evaluation, involving the processes of finding, selecting, synthesising, and interpreting data featured in documents [19]. Additionally, documents can be analysed to verify the findings.

In-pair, in-depth interviews facilitate more cohesive data collection, significantly surpassing the use of individual interviews when forming natural pairs [20]. In-pair, in-depth interviews were suitable for the participants due to their stable co-working processes as student groups and mentoring teams, trust in participation, and confidence in their interactive relationships. Four interviews—three in-pairs and one single interview—were conducted in this study. To strengthen the validity of the mentors' reports, we triangulated the mentors’ written reports of the mentoring programme for the document analysis.

### 2.1 Preunderstanding of data collector characteristics

The interviewers and facilitators were employees of the university. Transcription was performed by the first author, and the analysis was discussed with all four authors. The third author supervised the mentoring programme.

### 2.2 Sampling and recruitment

The empirical materials used in this study comprised interview data and written mentoring reports, as presented in Table 1. Eight students aged 27–55 years participated in the study (Table 2), including three male and five female students. Participants—both students and mentors—were purposively recruited from the mentoring programme at a university in Norway. All first-year students (N = 61) in a part-time nursing class with a mentoring programme were invited to participate. Recruitment for interviews took place between June 2022 and January 2023. Invitations were sent via email, and only four students agreed to participate. Reasons for non-participation were not thoroughly explored but may be attributed to a lack of time to being a part-time student, with family and work obligations in addition to school activities.

**Table 1:** Participants classified into interview groups/reports.

	Interview 1	Interview 2	Interview 3	Interview 4	Mentor reports	Total
Number of students (n = 4)	2	2				4
Number of Mentors with reports (n = 3)			2	1		3
Number of mentor reports (n = 1)					1	1
N = 8	2	2	2	1	1	8

**Table 2:** Participant characteristics.

	Students	Mentors
Sex		
Female	3 (75 %)	2 (50 %)
Male	1 (25 %)	2 (50 %)
Age		
Range	27–44 years	30–55 years
Mean	35,25 years	41 years
Median	35 years	39,5 years

Five students served as mentors in the online mentoring programme in nursing. Of these, three consented to be interviewed, and four consented to include their written reports. The fourth student could not participate in interviews due to pregnancy but consented to the use of her mentoring reports. The fifth student did not provide consent for either interviews or reports due to work commitments.

### 2.3 Data collection

Data were collected between June 2022 and January 2023. The digital platform Zoom was used for interview meetings due to the long distances between students and mentors in this part-time digital nursing programme. Zoom served as a meeting forum but did not facilitate recording. An audio recorder was used to capture the sounds from the computer, in accordance with ethical guidelines. The moderator took field notes during the interviews to trace conversations. These notes were analysed by the interviewer and moderators after the interviews and were secured and evaluated to understand the interviewing process. Interviews were conducted by the first author, with different moderators. The second and third authors served as moderators for interviews with students and mentors, respectively. The interviews followed semi-structured guidelines shown in Table 3. Interviews lasted from 30 to 75 min per session. No one other than the participants and researchers was present during the interviews. No pilot test could be conducted due to limited access to participants, and the interviews were conducted in an exploratory manner to allow students to describe their experiences. In-pair, in-depth interviews were conducted to obtain different opinions, providing additional points of view for supporting or influencing each other [21]. According to Braun and Clarke [18,22], thematic analysis is useful for working within a participatory research paradigm, with participants as collaborators. Data saturation was discussed among the authors and was concluded to be fulfilled, as the participants answered the semi-structured guide, meeting the thematic analysis criteria for the shared meaning of similarities and differences across the dataset. Repeated interviews would not provide any new or complementary information for the study, as the mentors only interacted with the same group of participants. Neither students nor mentors commented on the transcript but were asked to summarise their opinions at the end of the interviews. Participating students and mentors were invited to provide feedback on the findings during a meeting where the results of the study were presented.

**Table 3:** Interview guide.

Students	Mentors
1 Age/sex/study years or former work experience of the student 2 How did you experience mentoring this academic year? 3 How did you manage mentoring with your study time and work-life? 4 How is mentoring digitally? 5 How do you think mentoring in nursing field should be conducted?	1 Age/sex/study years or former work experience of the mentor 2 What were your expectations for mentoring? 3 How did you experience mentoring this academic year? 4 How did you manage mentoring with your study time and work? 5 How is it doing mentoring digitally? 6 How do you think mentoring in nursing field should be conducted? 7 How can universities implement mentoring?

### 2.4 Ethical considerations

This study was approved by the Norwegian Centre for Research Data (SIKT) (registration no. 225803) and conducted in accordance with ethical guidelines, following the principles of the Declaration of Helsinki of the World Medical Association [23]. All participants voluntarily provided informed consent, and the researchers ensured the anonymity of the data. Participants were informed of their right to withdraw consent at any time during the research period. They received information about the recording procedures, and the recorded files were securely sent directly to the first and second authors using the Dictaphone at the University of Oslo. All transcript copies were stored on a password-protected private computer. Participant names were converted into serial numbers to ensure anonymity.

### 2.5 Data analysis

**Fig. 1:**
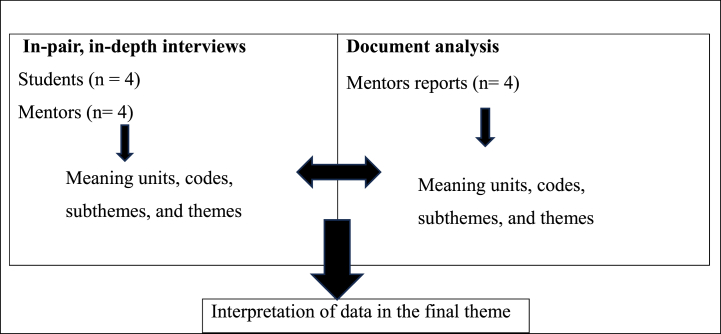
Flow chart of data analysis.

Data were analysed using the thematic analysis method described by Braun and Clarke [18,22], following the six recommended steps for data collection, with findings verified according to Bowen [19], as shown in Fig. 1. Themes were derived from the datasets through a stepwise process of data derivation: 1) The first, second, and third authors analysed the data by listening to the recorded audio, transcribing the data, and thoroughly reading the manuscript. 2) Data from each interview were then summarised and systematically classified into meaning units and potential code groups. The data were coded by the first, second, and third authors, who worked individually on the data material from both interviews and mentor reports. Written reports were systematically read for document analysis, identifying meaning units and codes for developing or confirming new subthemes. Bowen [19] recommends an iterative process involving skimming, reading, and interpretation, leading to thematic analysis of emerging themes that can be considered as categories. The documents were read individually, subthemes were developed, and themes were confirmed. 3) The first, second, and third authors then discussed the meaning units and patterns, settling on meaning units and code groups and framing potential subthemes, as shown in Table 4 (example of the interview analysis) and Table 5 (example from the document analysis). 4) The first and second authors then systematically reviewed the dataset again to include or exclude more meaning units for coding and generating a map of themes and subthemes. 5) Multiple research meetings were conducted among all four authors to continuously analyse, discuss, and negotiate the clarity of themes. The main themes were identified and discussed among all four authors. 6) Finally, the main themes were examined to state the final themes before the sixth step, completing the three final themes, writing the results, and final discussions based on the theoretical views.

**Table 4:** Example of the interview analysis.

Meaning units	Coding group	Subthemes	Themes
‘I thought we had a good approach and was excited to get started. And but then it collapsed, so we probably killed a lot of the engagement in the beginning’. (Mentor 1) ‘I thought the program in advance was really good, but then it just did not work out. I think it was overwhelming for students. Our mentoring was too offensive, maybe we scared away students with our program’. (Mentor 3) ‘At first, it was very overwhelming and it took some time to get to know each other and get started’. (Student 2) ‘We didn't have time’. (Student 3)	Not having equal interest and having a different starting point Disturbing and overpowering Overwhelming the students A lack of time for mentoring	‘Overwhelming Submission’ ‘Unlike interest or motivation’	‘**Differences in motivation**’

**Tabel 5:** Example from the document analysis.

Question	Meaning unit	Coding group	Subtheme	Theme
Q1: Describe mentoring process	‘Personally, I think that mentoring is a fantastic idea, not least in a digital way. Although nursing education is getting more and more digital, we are facing major challenges because of epidemics and pandemics’. (Mentor 2) ‘I think the mentoring has potential and can be very useful for the new students. However, students are unsure of what a mentor can do, so maybe the role of a mentor is a bit unclear ’. (Mentor 3) ‘When I joined as a mentor, I thought mentoring was an exciting project, I still think so, and I think it can actually be of great help to new students’. (Mentor 3)	Believing in mentoring ideas Potentials, but the role is unclear Exciting project for helping students	‘Motivated mentors, but unclear role’	‘**Strong motivation**’

## 3 Results

The overall peer mentoring experiences of both students and their mentors were categorised into three themes: ‘Differences in Motivation,’ ‘No Need for Digital Socialising,’ and ‘The Importance of Clarifying Organisational Tasks.’

### 3.1 Theme one: differences in motivation

Students and mentors had different starting points. The mentors were organised into teams of five, having completed a mentoring programme at the university. These mentors had themselves been students during the COVID pandemic, starting with few social meetings and less active relationships, thus expecting that new students might need help integrating into student groups. However, mentors were surprised when the students did not show interest in them and felt they were disturbing the students by sending emails, which often went unanswered.

‘We tried to get in touch with students several times, and then there was either no one, or maybe one or two who contacted us. We then replied to them, but we did not receive any response. It was a bit awkward, I have to say.’ (Mentor 1)

‘We were very excited, and then we did not get the response we expected.’ (Mentor 2)

The students reported a lack of time to get to know the mentors and did not understand the objective of being assigned mentors. They reflected on their inability to prioritise contacting the mentors due to the stress of being freshers.

‘I found it a bit difficult to understand the role of the mentors.’ (Student 4)

‘We did not have time. We talked to him once. Maybe we prioritised completely wrong?’ (Student 3)

### 3.2 Theme two: no need for digital socialising

Students fulfilled their social needs through interactions with each other rather than with mentors. Their capacity to socialise was limited by a lack of time or difficulty in prioritising it. The mentors realised that the students did not have social needs related to their studies, often due to family obligations or work commitments alongside their education.

‘I think the student study group we are assigned to is sufficient. We have our own channels where we arrange to meet; it was very straightforward.’ (Student 1)

‘The student group was easy to integrate into, using internal social media like chat groups and Zoom. We supported each other well, both for technical support and social interactions.’ (Student 3)

‘I guess most students are “grown-up,” and the obligations they have besides studying, in terms of work, family, etc., make a big difference.’ (Mentor 1)

The student groups had internal fellowships and did not require mentors for support. Additionally, they provided technical and social support to each other. Regarding the social component, both mentors and students found digital socialising unnatural and suggested a better academic focus. Students and mentors emphasised the importance of more professional relationships instead.

‘It’s okay when the mentors just join the meeting, like when they are just in the group to check, but it was difficult to make an appointment or anything like that with them. Their involvement, such as in medical mathematics, through Zoom was helpful, it really was. But otherwise, it was difficult to achieve anything with them in the student group.’ (Student 2)

‘We all felt it was very unnatural, and why would we socialise digitally? I think it would be more concrete to help them with anatomy or something like that. For example, the topic they are studying in that particular week, then they may feel more need of us.’ (Mentor 3)

### 3.3 Theme three: the importance of clarifying organisational tasks

Digital peer mentoring was not perceived as a valuable arrangement in the nursing programme. The mentors had difficulty contacting the students and expressed frustration, feeling as though they were nagging. When the students did not respond, the mentors experienced personal uncertainty.

‘You felt a little bit like I had done too little. I even started to be unsure of myself at the end of the year—not directly, but it was kind of that weird feeling. Have I written something wrong? Have I misrepresented myself? What have I not done right? No feedback, right, so that was it?’ (Mentor 2)

However, students desired more guidance from their mentors and felt uncertain when they had to initiate an appointment and specify what they needed help with. They preferred to rely on the university for more direct instructions to align with their agendas and set time schedules.

‘It was really good to have them in digital class when they just popped in and asked how we were doing, how we felt, what they did last year, and all that.’ (Student 4)

‘I think we should have been a little more included in the digital teaching itself, so they got to know us a little bit better.’ (Mentor 1)

‘I got the impression that the students felt a need for extra academic input and help with academic writing. You must work on subjects; you have to be given specific assignments.’ (Mentor 3)

‘For me, having the feeling of being able to contribute to something, which I got through the mentoring task in medical calculation classes, was important. That meant I did not withdraw from the whole project.’ (Mentor 2)

## 4 Discussion

This study presents the findings of digital peer mentoring in a nursing programme. The outcomes reveal that mentors have difficulties maintaining digital peer mentoring with the students and that students express little need for online mentoring. The literature on mentoring specifies how new students need peer support from mentors to clarify the academic curriculum, and mentors being more accessible than teachers contribute to students' social relations and help alleviate academic stress ([5]; [4]; [2,7,8]). However, both students and mentors found the mentoring process difficult in this study. Although mentors are obligated to proceed with the mentoring programme, students can decline participation because it is voluntary for them. These baseline dissimilarities may contribute to a difference in motivation between students and mentors. Moreover, mentors could not support students’ needs, and students did not acknowledge the supportive role of their mentors. As the learning process is facilitated in a socially situated placement [6], a virtual placement may not have the same stability as a physical placement or may exhibit different demands. Therefore, online learning is different from traditional learning [15], as tutoring online classes requires clear directions and specific goals for active utilisation [24]. Students not contacting their online mentors is paradoxical because a virtual context should theoretically provide more opportunities for spontaneous or ad hoc meetings than physical settings in university timetables and schedules.

Students in this study rejected their online peer mentors, finding online digital support within their own class. Salmon [25] reported that students are now more digitally competent and familiar with online systems, enabling them to support each other easily when connected online. Students often form class groups on social media, where information is shared and classroom issues are discussed virtually after classes, spreading information quickly to a larger group and reducing or eliminating the waiting time for email responses from mentors or teachers. The students stated that assigning mentors was an unnecessary service for them, as their interaction with fellow students was more helpful in managing academic stressors. Part-time nursing students may face different challenges in the context of mentoring compared with on-campus nursing students due to time constraints or difficulty prioritising. Part-time nursing students often work part-time alongside their studies, with more family obligations significantly impacting their study time compared to students in full-time programmes.

Surprisingly, for mentors, peer mentoring was a challenging experience, involving struggles with personal integrity. Moreover, they doubted their online mentoring social skills when the students rejected their services. Adequate social skills are imperative for performing in the professional nursing field. Failing in the mentor setting, from the mentor's point of view, can be similar to lacking these necessary social skills. The mentors in this study began to develop self-doubt but found comfort in the mentor group as well as with their mentor programme supervisors. The psychological team-building ability of mentors is crucial in mentoring programmes, owing to barriers in initiating meetings and maintaining contact with students [10].

**Fig. 2:**
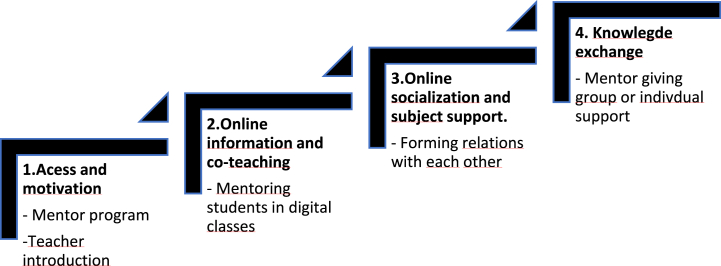
Online mentoring ladder.

In this study, both students and mentors expressed more success when mentors were assisting teachers. They were satisfied with mentoring in direct academic work during online classes, for instance, when mentors and teachers co-taught medical mathematics. Facilitation in online classes resulted in more legitimacy for peer mentoring than constructing their own online environment. In constructive alignment, the learning process is a result of student activities [26]. The alignment results in a didactical threefold relationship between the teacher, the content of assessments, and the students [27]. The choices teachers make in assessments are fundamental to achieving alignment. Therefore, teachers are considered gatekeepers of student interactions [10]. Mentors can collaborate more with teachers in online classes and engage in inter-relationships with the students. Scaffolding learning processes [28] can be executed digitally through didactic alignment planned by teachers. Based on Salmon's five-step relational model (2013), digital online mentoring needs more ‘onboarding’ facilitation and teacher support, as shown in Fig. 2.

The online learning perspective of Salmon's theory (2013) refers to social constructionism and an interpretive paradigm. Constructive theory explains the learning process as a constant change in perspective, forming personal interpretation and establishing understanding [27]. Scaffolding learning indicates that students' knowledge and personal development can expand when supported by mentors, and it also provides a new dimension to student mentors. Therefore, mentoring programmes are important for the personal and professional development of both students and mentors [7,11]. Collaborative learning processes through constructive alignment are useful for arguing that social interaction and communication are necessary for learning, explaining why certain views are constructed. Social constructivism research seeks to understand how individuals interact within their environment and biographies and how this is rooted in a sociocultural context [21]. Connecting and adapting students to the online environment are crucial for constructing a digital part-time nursing programme.

Universities should facilitate the mentoring process, as mentors' activities may be systematically included in the educational quality systems [29], providing more awareness to faculty and an advisable structure for mentoring programmes [30]. Better availability and accessibility are advantages of assigning peer mentors to students [8], thereby helping students develop their identity at the university rather than relying solely on faculty staff mentors [31]. Enhanced organisational arrangements for co-working make the didactical threefold teacher-mentor-student relationships more effective, preventing mentor barriers. After familiarising themselves with co-teaching and serving in academic settings, peer mentors may also contribute to meeting students' social needs by building trust in academic relationships. In terms of social skills in nursing, peer mentoring processes can strengthen mentors’ personal integrity, provide guidance and supervision in mentoring teams, and advance programme development. Therefore, an academic interpretation is necessary.

## 5 Conclusion

The findings of this study can contribute to new organisational perspectives on peer mentoring in digital settings for nursing programmes. Universities need a more reflective approach towards collaborative learning processes to achieve student learning outcomes, where the formalisation of mentoring programmes can improve the quality of peer mentoring activities in higher education. Furthermore, mentors must be systematically included in the educational quality systems to be a fruitful resource for peer mentoring. Moreover, conceptualising a formal mentoring programme based on qualitative evaluations of students and mentors may provide adequate results. Faculty awareness and specified guidelines for peer mentoring in higher education could potentially enhance the success of online mentoring programmes in nursing.

## CRediT authorship contribution statement

**Hilde Elisabeth Toreid:** Conceptualization, Data curation, Formal analysis, Methodology, Project administration, Validation, Visualization, Writing – original draft, Writing – review & editing. **Berit Harriet Mosseng Sjølie:** Conceptualization, Data curation, Formal analysis, Methodology, Validation, Writing – original draft, Writing – review & editing. **Sverre Andreassen Bjørbæk:** Data curation, Formal analysis. **Marcel Köhler:** Validation, Visualization, Writing – review & editing.

## Limitations

This study was conducted in Norway and may exhibit Western and Nordic bias. However, similarities can be observed with studies conducted in Eastern countries, such as the study by Kang et al. [24]. More studies from different regions would enhance our understanding of online peer mentoring processes in higher education. Furthermore, the sample size of this present study was small due to the limited number of mentors in one nursing mentoring programme, thus the results may not be generalisable to mentors in other nursing programmes.

## Funding

This study did not receive any specific grants from funding agencies in the public, commercial, or non-profit sectors.

## Declaration of competing interest

The authors declare that they have no known competing financial interests or personal relationships that could have appeared to influence the work reported in this paper.
